# Rationale and design of the HepZero study: a prospective, multicenter, international, open, randomized, controlled clinical study with parallel groups comparing heparin-free dialysis with heparin-coated dialysis membrane (Evodial) versus standard care: study protocol for a randomized controlled trial

**DOI:** 10.1186/1745-6215-14-163

**Published:** 2013-06-01

**Authors:** Patrick Rossignol, Marc Dorval, Renaud Fay, Joan Fort Ros, Nathalie Loughraieb, Frédérique Moureau, Maurice Laville

**Affiliations:** 1 INSERM, Centre d’Investigations Cliniques 9501, Institut lorrain du Cœur et des Vaisseaux Louis Mathieu, 4 Rue du Morvan, 54500 Vandoeuvre lès Nancy, France; 2 Université de Lorraine, Lorraine 54500 Vandoeuvre lès Nancy, France; 3 CHU de Nancy, Nancy 54500 Vandoeuvre lès Nancy, France; 4 INSERM U1116, 54500 Vandoeuvre lès Nancy, France; 5 Association Lorraine pour le Traitement de l’Insuffisance Rénale, 54500 Vandoeuvre lès Nancy, France; 6 Centre hospitalier universitaire Dr-Georges-L-Dumont, Moncton NB E1C 2Z3 Canada; 7 Nephrology Department, Dialysis unit, University Hospiital Vall d'Hebron, Paseo Vall d’Hebron 119-129, 08035 Barcelona, Spain; 8 Autonomous University of Barcelona, Barcelona, Spain; 9 Gambro-Hospal, 69881 Meyzieu, France; 10 Service de Néphrologie, Centre Hospitalier Lyon-Sud, 69495 Pierre-Bénite, France; 11 Université Lyon 1, 69100 Villeurbanne, France; 12 Inserm U1060-Institut CarMeN, 8 Avenue Rockefeller, 69373 Lyon, France; 13 Association pour l’Utilisation du Rein artificiel à Lyon (AURAL), 124 Rue Villon, 69008 Lyon, France

**Keywords:** Hemodialysis, Heparin-free, Evodial, Randomized controlled trial

## Abstract

**Background:**

Anticoagulation for chronic dialysis patients with contraindications to heparin administration is challenging. Current guidelines state that in patients with increased bleeding risks, strategies that can induce systemic anticoagulation should be avoided. Heparin-free dialysis using intermittent saline flushes is widely adopted as the method of choice for patients at risk of bleeding, although on-line blood predilution may also be used. A new dialyzer, Evodial (Gambro, Lund, Sweden), is grafted with unfractionated heparin during the manufacturing process and may allow safe and efficient heparin-free hemodialysis sessions. In the present trial, Evodial was compared to standard care with either saline flushes or blood predilution.

**Methods:**

The HepZero study is the first international (seven countries), multicenter (10 centers), randomized, controlled, open-label, non-inferiority (and if applicable subsequently, superiority) trial with two parallel groups, comprising 252 end-stage renal disease patients treated by maintenance hemodialysis for at least 3 months and requiring heparin-free dialysis treatments. Patients will be treated during a maximum of three heparin-free dialysis treatments with either saline flushes or blood predilution (control group), or Evodial. The first heparin-free dialysis treatment will be considered successful when there is: no complete occlusion of air traps or dialyzer rendering dialysis impossible; no additional saline flushes to prevent clotting; no change of dialyzer or blood lines because of clotting; and no premature termination (early rinse-back) because of clotting.

The primary objectives of the study are to determine the effectiveness of the Evodial dialyzer, compared with standard care in terms of successful treatments during the first heparin-free dialysis. If the non-inferiority of Evodial is demonstrated then the superiority of Evodial over standard care will be tested. The HepZero study results may have major clinical implications for patient care.

**Trial registration:**

ClinicalTrials.gov NCT01318486

## Background

Hemodialysis treatment requires anticoagulation, usually with unfractionated or low molecular weight heparin to prevent thrombosis of the dialyzer and the extracorporeal circuit (dialyzer and blood lines or cassette system). In clinical practice, it is not unusual to perform hemodialysis for patients with active bleeding or increased bleeding risk conditions when heparin anticoagulation is contraindicated. Various solutions have been attempted to prevent clotting of the extracorporeal circuit.

Heparin-free dialysis using intermittent saline flushes is widely adopted as the method of choice for patients at risk of bleeding [1,2] and is currently recommended by the European Best Practice Guidelines in hemodialysis (2002) [3], although the level of evidence is somewhat weak since no randomized controlled studies evaluating this method have been reported to date [4-11]. Heparin-free treatment with on-line predilution rather than regular saline flushes is also a technique used at some dialysis units. Saline infusion is far from optimal for several reasons, including an increased volume load that would need to be removed with dialysis and an added logistic burden on dialysis nurses, owing to the need for close one-to-one nursing in cases of intermittent boluses [1]. In addition, this technique is still associated with clotting risks (15% to 35%, depending on the literature) which, in addition to blood loss, may reduce the efficiency of a dialysis session that has to be stopped frequently or prematurely. Regional (heparin administration into the arterial line and protamine into the venous line) or tight heparinization (use of minimal dose of heparin) is not recommended for patients with active bleeding or at risk of bleeding [3]. Indeed, although these methods lower the risk of bleeding compared to the standard method, there is still a notable risk of bleeding (5% to 50%) [12].

The use of regional citrate anticoagulation (RCA) is limited by the need for additional equipment (additional pumps for citrate and calcium), the potential risk of deterioration of electrolyte and acid–base equilibrium (hypocalcemia, hyponatremia) requiring close monitoring of electrolytes [12], and a need for clotting time evaluation since the risk of bleeding is reduced but may still occur. Recently, the feasibility of anticoagulating the extracorporeal circuit during hemodialysis using a simple citrate-enriched dialysate was evaluated in a pilot (no prespecified sample size calculation or primary endpoint), monocentric, prospective, randomized, crossover study of 24 patients with high risk of bleeding [13]. For anticoagulation of the extracorporeal circuit, one treatment used the citrate-enriched dialysate (citrate group), while the other treatment used conventional saline flushing (saline group). With either method, a heparinized, saline-rinsed polyamide dialyzer was used and no heparin was administered during dialysis. Ninety-two per cent (22 out of 24) and 100% of patients tolerated the procedure well in the citrate group and saline group, respectively. Eight per cent (two out of 24) of the treatments in each group had to be abandoned because of clotting in the extracorporeal circuit, while significantly less thrombus formation in the venous air traps was detected in the citrate group [13]. Other alternatives such as prostacyclin are costly and require close hemodynamic monitoring [3]. In a pilot (without sample size calculation or prespecified primary endpoint), monocentric, randomized, controlled trial, Yixiong *et al*. recently compared heparin-free saline flushes with low-dose argatroban (a direct thrombin inhibitor) in a total of 80 hemodialysis sessions performed in 52 patients with high risk of bleeding. They observed a three-fold decrease in major clotting with argatroban (*P* <0.05), without safety issues [14].

Another alternative for heparin-free hemodialysis is to coat heparin on the hemodialyzer hollow fibers [1,12]. Stamatiadis *et al*. reported the results of 16,954 sessions performed with patients bleeding or at risk of bleeding (15,730 retrospectively and 1,224 prospectively collected), with a method combining a priming of two types of hollow fiber wet dialyzers (ethylene vinyl alcohol and polyethylene glycol-coated membranes with 1 liter of saline containing 5,000 IU of unfractionated heparin). After initiation of the treatment, except for hourly rinsing with 50 ml normal saline to inspect the circuit (100 ml for a 3-hour session), no other special nursing attendance was applied. Cumulative failure of treatment, as defined by clotting of the extracorporeal circuit requiring termination of the procedure or replacement of the clotted part, was not greater than 5% [15].

Gambro (Lund, Sweden) have for several years developed coated hemodialyzers such as Nephral ST that are equipped with a membrane, the surface of which is treated with polyethyleneimine (PEI) allowing extemporaneous coating with heparin when the hemodialyzer is primed with saline solution containing heparin. Several clinical studies have been performed with Nephral ST to demonstrate the possibility of decreasing systemic heparin doses or of even performing hemodialysis treatments without heparin [16-18]. In a study aimed at evaluating the AN69 ST membrane in heparin-free dialysis in patients at risk of bleeding (n = 68) less than 2% massive clotting were observed (6 out of 331 dialysis sessions) [17]. Evenepoel *et al*. performed a monocenter, prospective, randomized trial including 33 hemodialysis patients at high risk of bleeding, in whom regional anticoagulation was achieved by means of either AN69 ST (Nephral ST; 11 patients, 31 sessions), RCA-Ca0 (11 patients, 32 sessions) or RCA-Ca3.0 (11 patients, 30 sessions). Clotting phenomena necessitating premature termination of the dialysis session were encountered in 39%, 13% and 0% using AN69 ST, RCA-Ca3.0 and RCA-Ca0, respectively (*P* <0.005). The authors concluded to the superiority of citrate regional anticoagulation [19]. In a bicentric, Austrian, randomized, crossover trial, Kodras *et al*. treated 10 patients receiving oral anticoagulants with AN69 ST and FX 100 (a polysulfone membrane) for 1 week (total 30 sessions of heparin-free dialysis, with only priming of the dialyzer and no systemic heparinization). Changes of clotting markers, occurrences of complete thrombosis and Kt/V were similar with both membranes [20]. More recently, a prospective, randomized, crossover study examined 10 stable patients during intermittent hemodialysis with: 1. regular saline flushes of extracorporeal circuit; 2. RCA; and 3. AN69 ST membrane after extracorporeal circuit priming. All 10 procedures with RCA were successfully completed after 4 hours, whereas six out of 10 procedures with saline flushes and five out of 10 procedures with AN69 ST were terminated prematurely because of clotting (*P* <0.05). Due to insufficient statistical power of the negative results, the number of incomplete procedures did not allow the authors to directly compare saline flushes and AN69 ST [21].

A new dialyzer, Evodial (Gambro), which is an upgrade of the Nephral ST dialyzer, is grafted with unfractionated heparin during the manufacturing process. *In vitro* and *in vivo* data requested for CE marking have shown the stability of heparin grafting. Several clinical studies have been performed with Evodial [22,23]. A 30% heparinization reduction with Evodial dialyzers led to an improvement in oxidative stress, thereby testifying an effective biocompatibility [22]. In a study performed in 45 chronic dialysis patients, Kessler *et al*. have shown that the systemic heparin dose, regardless of heparin type (unfractionated heparin or low molecular weight heparin), can be reduced by 45% ± 13% without any coagulation issues, which was defined as very early clotting signs such as quality of rinse-back in the circuit (dialyzer and blood lines) [18].

The present HepZero study hypothesis is that in patients requiring heparin-free dialysis, the heparin-free treatment with Evodial can be easily performed (without saline flushes or blood predilution) and is at least not inferior and may be superior to the standard care heparin-free treatment in terms of clotting, as assessed for the first time by an international, multicenter, randomized, controlled, open-label trial with two parallel groups.

## Methods/Design

### Study design

**Figure 1:**
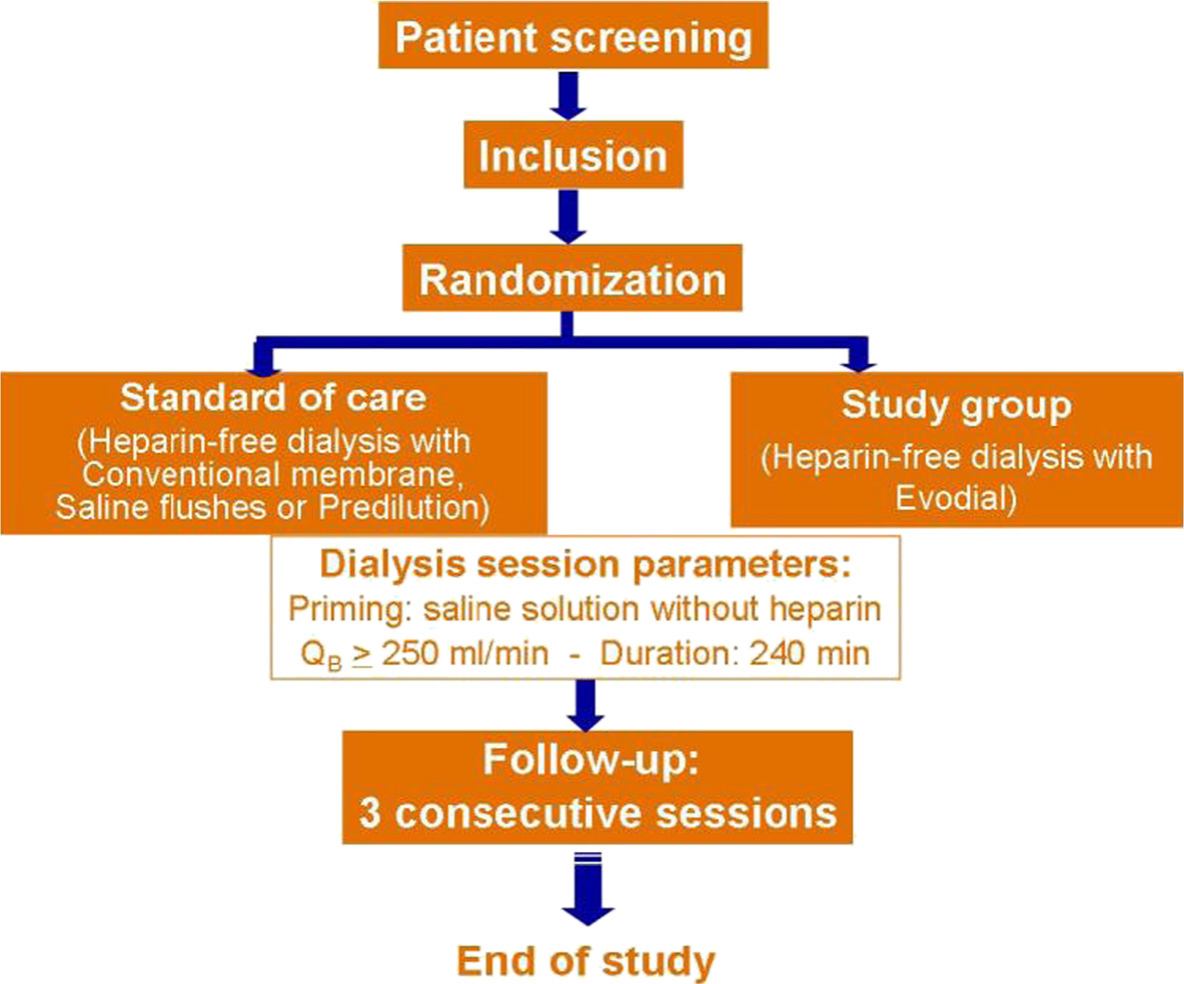
Study flow-chart.

This clinical study is a prospective, multicenter (10 centers), international (7 countries), open, controlled, randomized clinical study (Figure 1). Two types of therapies will be evaluated in parallel.

The control group will receive heparin-free hemodialysis treatment according to standard care. Since there is no unique standard heparin-free treatment, the control group will receive the usual procedure in place at each study site (saline flushes or predilution) with guidelines aimed at standardizing practices within the control group. The standard care can be either saline flushes during dialysis treatment (100 ml to 300 ml per flush every 30 minutes, as stated in the European Best Practice Guidelines (2000) [3]) or predilution (on-line or bags, with a predilution rate between 1 l/h and 2 l/h; high-volume hemodiafiltration is not allowed).

The study device group will receive heparin-free hemodialysis treatment with Evodial. To allow the comparison, a dialyzer with roughly the same surface area as Evodial 1.6 (1.65 m^2^) will be used in the study group. Consecutive patients will be screened by the investigators and, when eligible, will be enrolled in the study and treated during a maximum of three consecutive heparin-free dialysis treatments, without any switch allowed between arms.

#### Primary objectives

The primary objectives of the study are to determine the effectiveness of the Evodial dialyzer, compared with standard care in terms of successful treatments during the first heparin-free dialysis. If the non-inferiority of Evodial is demonstrated then the superiority of Evodial over standard care will be tested.

#### Secondary objectives

The secondary objectives are: to compare the success rate during the second and third consecutive heparin-free dialysis treatment with Evodial to standard care; to compare clotting grades in air traps during all treatments with Evodial versus standard care; to assess the efficacy of heparin-free dialysis treatment with Evodial versus standard care; to assess the ease of use of heparin-free dialysis treatment with Evodial; and to follow-up the safety of heparin-free dialysis treatment with Evodial versus standard care.

#### Study population

The patients to be included in this clinical study are end-stage renal disease patients treated by maintenance hemodialysis for at least 3 months and requiring heparin-free dialysis treatments. Reasons for heparin-free dialysis prescription will be recorded and may include: gastrointestinal hemorrhage; invasive procedure: pre-, post-procedure; perioperative: pre- or post-surgery; cerebral hemorrhage: type and date; other bleeding risk; other reason not related to bleeding risk; cholesterol emboli; diabetic retinopathy; and other reason.

Bleeding risk will be graded according to the Lohr and Schwab definition [24]: very high risk, active bleeding at time of dialysis; high risk, active bleeding stopped for less than 3 days or surgery or trauma within the previous 3 days; moderate risk, active bleeding stopped for more than 3 days but less than 7 days, surgery or trauma within the previous 3 to 7 days, or uremic pericarditis or pleuritis; and low risk, greater than 7 days after active bleeding, surgery or trauma.

These patients shall meet the following inclusion and exclusion criteria defined for this study. Inclusion criteria are the following: patients requiring heparin-free dialysis treatments as per nephrologists’ prescription; chronic end-stage renal disease patients treated by maintenance hemodialysis for at least 3 months; patients with a well-functioning blood access that can allow a blood flow of at least 250 ml/min; patients aged 18 years or older;written consent to participate in the study (informed consent).

Patients that have already been treated with heparin-free hemodialysis can be included into the study. The first treatment is defined as the first treatment evaluated when the patient is enrolled in the study. Patients with a catheter locked by heparin can be included in the study, although particular attention must be paid to the removal of heparin and the rinsing of the catheter prior to starting the hemodialysis treatment.

Exclusion criteria are the following: patients in intensive care unit settings; acute kidney injury patients; patients dialyzed in self-care, satellite hemodialysis units; patients treated in single needle mode; known heparin contraindication (heparin-induced thrombocytopenia type II); patients requiring blood and other labile blood products (for example fresh frozen plasma, platelets) transfusion during hemodialysis treatment; patients receiving oral anticoagulants (including anti-vitamin K); patients receiving a combination of anti-platelet agents; patients treated with unfractionated or low molecular weight heparin in addition to the dialysis treatment to prevent deep vein thrombosis; pregnant/planning pregnancy and lactating women during the study period; legally-protected adult patients; patients not affiliated with a health insurance system (beneficiary or dependent); participation in other interventional studies during the study period; and patients that have already been included in this study.

Patients will be considered enrolled in the study when the informed consent has been signed. Time zero is when the randomization is done. Withdrawn patients will be replaced only if the patient has been randomized and no dialysis treatment could have been performed or in case of an improper second enrolment of the same patient (the second one being not considered).

### Study endpoints

#### Primary endpoints

**Figure 2:**
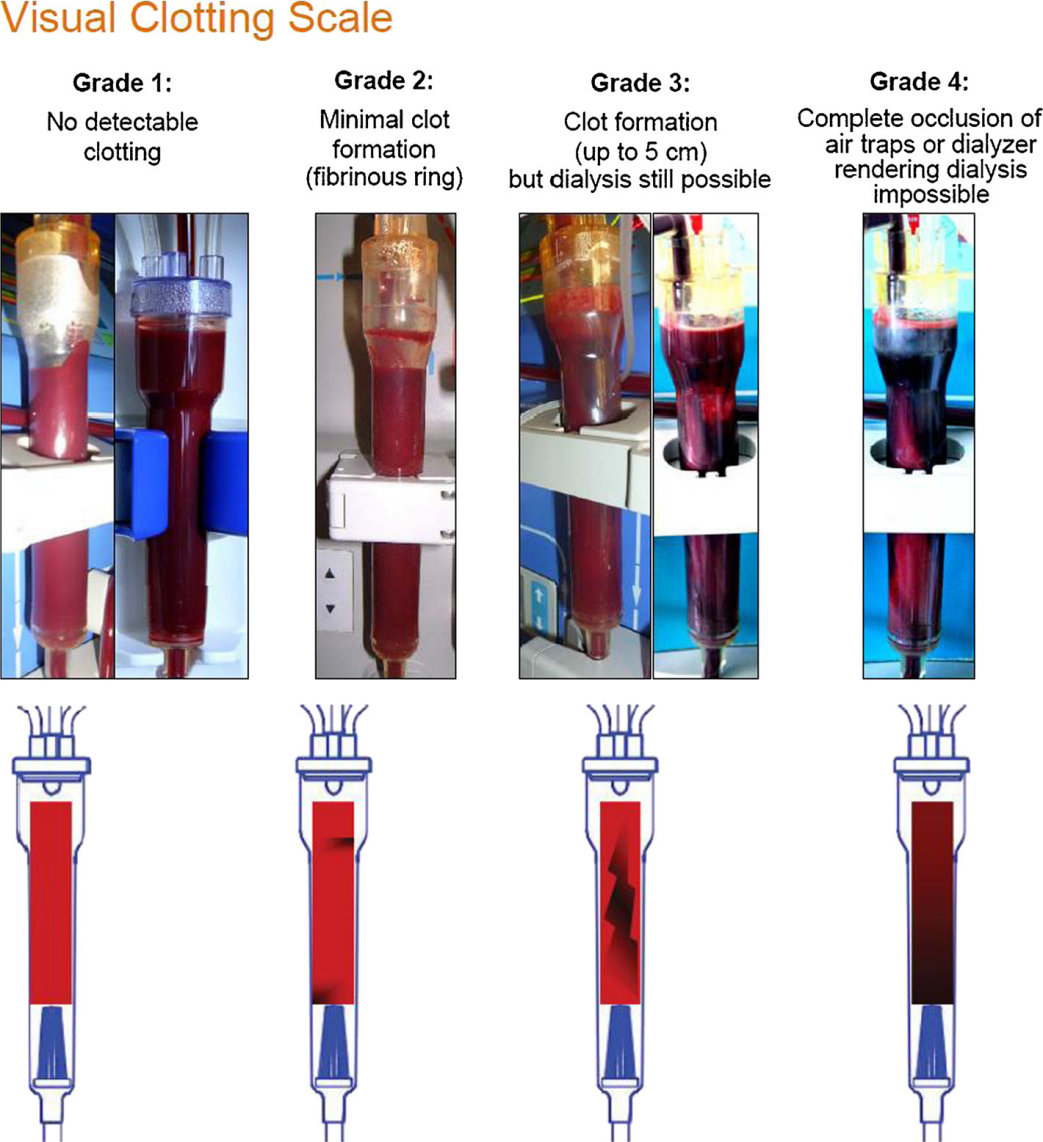
Visual clotting scale.

In order to evaluate the rate of successful treatments, clotting in the air traps will be scored using a semi-quantitative scale (Figure 2) [13,14,19,20,25,26]: grade 1, no detectable clotting; grade 2, minimal clot formation (presence of fibrinous ring); grade 3, clot formation (up to 5 cm) but dialysis still possible; and grade 4, complete occlusion of air traps or dialyzer rendering dialysis impossible.

The first heparin-free dialysis treatment will be considered successful when there is: no complete occlusion of air traps or dialyzer rendering dialysis impossible (grade 4); no additional saline flushes to prevent clotting; no change of dialyzer or blood lines because of clotting; and no premature termination (early rinse-back) because of clotting.

This evaluation (clotting scoring) will be performed hourly by two independent observers. Depending on the organization at each study site, the evaluation can be performed by two nurses (the nurse in charge of the patient and a second nurse not in charge of the patient) or by the nurse in charge of the patient and a co-investigator. In case of discordance between the two observers or in the eventuality of premature session termination (grade 4), the final decision will be made by a third authorized and trained person (the principal investigator or registered co-investigators).

At each site and prior to the enrolment of patients, an organization will be implemented, and the investigator and all those involved in the study will be trained and certified with regard to scoring.

### Secondary endpoints

The follow-up of the clotting during the first heparin-free dialysis treatment will be performed at each hour throughout the dialysis session; clotting in the air traps will be assessed using the same semi-quantitative scale as described above.

The evaluation of the success rate during the second and the third consecutive heparin-free dialysis treatments will be as for the first heparin-free dialysis treatment. Treatments will be considered successful when there is: no complete occlusion of air traps or dialyzer rendering dialysis impossible (grade 4); no additional saline flushes to prevent clotting; no change of dialyzer or blood lines because of clotting; and no premature stoppage (early rinse-back) because of clotting. This evaluation will be performed by two independent observers as described in the aforementioned primary endpoint section.

Follow-up of the clotting during the second and the third consecutive heparin-free dialysis treatment will be performed at every hour; clotting in the air traps will be assessed using the same semi-quantitative scale as described above.

To undertake hemodialysis session efficacy assessment, the ultrafiltration (UF) achieved, weight loss, serum creatinine and urea reduction rates, as well as electrolytes changes will be documented during all dialysis sessions. To measure ease of use, the frequency and remaining volume of saline flushes will be documented. The occurrence of adverse events and serious adverse events during the study will be collected.

### Ethics

Inclusion in the study was initiated in 2011 after approval of the appropriate ethics committee (Comité de Protection des Personnes, Sud Est III Lyon, France; Comité Ético de Investigación Clínica, Vall d’Hebron Hospital, Barcelona, Spain; Comité Ético de Investigación Clínica, Germans Trias i Pujol Hospital, Barcelona, Spain; National Research Ethics Service, Yorkshire and the Humber – Sheffield, UK; Comité d’éthique, Université Libre de Bruxelles, Hôpital Erasme, Brussels, Belgium; Comité d’éthique de la recherche Centre hospitalier universitaire, Dr-Georges-L-Dumont, Moncton, Canada; Medisch Ethische Toetsingsingscommissie, Universitair Medisch Centrum Groningen, Groningen, the Netherlands; and the Independent Bioethic Committee for Scientific Research, Gdańsk Medical University, Gdańsk, Poland) and competent authorities where applicable. The study protocol was recorded prior to any enrolment at ClinicalTrials.gov: NCT01318486.

### Statistics

Allocation of treatments will be performed using a centralized, on-line randomization system. Patients will be assigned to treatment groups using block randomization stratified on centers.

### Justification of sample size

The main criterion of efficacy is the success rate in the Evodial group (test) compared to standard care (on-line predilution or saline flushes). After completion of the study, the analysis will follow a two-step procedure and the probability that the efficiency of Evodial is inferior to that of the standard care will be tested first. If that probability is rejected at the 5% significance level, that is if non-inferiority is demonstrated, then superiority will be tested. The probability of simultaneously falsely accepting inferiority and superiority being mutually exclusive, no adjustment of alpha error rate will be requested. According to the results, the conclusion will be: Evodial is inferior, not inferior but not superior, or superior to the standard care.

According to the data published on heparin-free treatment (with saline flushes or on-line predilution), the success rate of hemodialysis ranges from 65% to 85%. Recent works presented at the French Society of Nephrology, September 2010, meeting in Brussels, showed a 15% to 20% improvement of these success rates using preheparinated dialysis membranes (HeprAN, Gambro, or AN69 ST) [27,28]. The non-inferiority as well as superiority margins will be set to the same value of 15%. In the hypothesis of a 65% success rate (the most patient-consuming) in the control arm, a sample size of 126 patients per arm (252 for the entire sample) will provide the trial with 80% power to first conclude to non-inferiority then to superiority, with a one-tailed 5% error rate. Assuming an equal inclusion rate in all centers, 26 patients will be enrolled in each of the 10 centers, with 13 patients in each arm. Withdrawn patients will be replaced only if the patient has been randomized and no dialysis treatment could have been performed.

### Efficacy analysis

#### Baseline comparability

The baseline comparability of groups will be checked, using the center as an adjustment factor. Should one or more factors show significant imbalance, the steering committee will assess the impact of the possible confounding factor(s) on further analyses and decide whether it is necessary to take it into account during the blind review of data. Adjustment will be performed using logistic regression.

#### Primary objective

The success of hemodialysis is defined as the proportion of first treatment achieved without prematurely halting dialysis or having to change blood lines or dialyzer, owing to clotting or any additional saline flush because of clotting. The 95% one-tailed confidence interval of the test-reference difference will be constructed using the Wilson method [29], and non-inferiority then superiority accepted if its lower boundary is found to be greater than −15% then +15%. The consistency of responses across centers will be confirmed using the Breslow-Day test for homogeneity of odds ratios or logistic regression as appropriate. Though unexpected, the impact of maneuvers in the control arm (predilution or saline flushes, centre-specific) will be tested and, if necessary, taken into account as a nested factor in the analyses.

#### Secondary objectives

The treatment groups will be compared for efficacy (success rates in second and third treatments, achievement of the planned weight loss and UF volume) and safety using the Mantel-Haenszel test stratified on center or logistic regression as appropriate.

#### Patient populations

The full analysis set will consist of all patients who received at least one treatment and had one efficacy assessment. Since the primary criterion is the success of the first treatment, no dropout is expected, and intention-to-treat and per-protocol populations will be identical except in case of deviations (for example not allowed medication or inappropriate hemodialysis modality with regard to the randomization arm).

### Study organization (in the Additional file 1 the steering committee, DSMB and HepZero investigators are listed)

#### Steering committee

In conjunction with the sponsor, a steering committee will oversee the trial. The steering committee is the main policy and decision-making committee of the study and has final responsibility for scientific conduct. The specific tasks of the steering committee are to: approve by signing the study protocol and protocol amendments; provide recommendations to solve problems in cooperation with the clinical study manager; and approve study reports and articles for publications (including abstracts) and presentation of clinical data of any investigator.

The steering committee is comprised of three investigators participating in this study, one methodologist and nephrologist (PR) and two representatives from the study sponsor, Gambro.

An independent Data and Safety Monitoring Board (DSMB) will monitor the safety and efficacy of the trial and periodically assess whether the trial should continue to the planned termination. Based on the safety data, the DSMB may recommend modifications to the protocol (for example amendments, termination of the study) and, when needed, the DSMB will decide on stopping rules. The DSMB will consist of three members. No member of the DSMB will act as an investigator for the study. Members of this board are not affiliated with Gambro, the principal investigators or the clinical investigation. They will declare any conflicts of interest if such should arise. The DSMB will report to the chairman of the steering committee, who in turn is responsible for implementing a decision to terminate the trial prematurely if deemed necessary.

## Discussion

Anticoagulation for chronic dialysis patients with contraindications to heparin administration is challenging. The regimens used as alternatives, for example anticoagulation-free dialysis, regional heparinization with protamine, direct thrombin inhibitors, prostacyclin or RCA, encounter considerable limitations. Current guidelines state that in patients with increased bleeding risks, strategies that can induce systemic anticoagulation should be avoided. Treatment strategies that avoid this include no use of anticoagulants with regular flushing or RCA [3]. To date, the use of RCA is restricted to specialized units, since its application is cumbersome [26], because it usually requires additional pumps for citrate and calcium infusions, which are not provided by standard dialysis machines, as well as the need for careful monitoring of electrolytes. These issues not only increase the complexity of the RCA procedure but also the likelihood of complications, the most dangerous being systemic hypocalcemia, since it can cause life-threatening arrhythmias [30]. Therefore, heparin-free dialysis using saline infusion is currently *de facto* considered as the gold standard in patients with high risk of bleeding [1,2] and serves as the control intervention in the present trial. For obvious logistical and technical reasons, the study is conducted as open-label and the primary endpoint could not be assessed blindly. Indeed, neither the predilution process nor the saline flushes can be masked and the dialyzers can easily be differentiated by the nursing staff (different housings, different membrane colors and transparency), since they must be carefully examined during the dialysis treatment and they cannot be completely covered by a label aimed at preventing any differentiation between the two groups. In order to minimize potential bias due to the open-label design, it was decided to have the study primary endpoint: 1. evaluated by the most widespread semi-quantitative clotting scale used in research [13,14,19,20,25,26]; 2. independently rated by two observers; in case of discordance between the two observers or in case of premature session stoppage (grade 4), the final adjudication must be made by a third authorized and trained person (the principal investigator or registered co-investigators); and 3. training and certification of nurses and investigators with regard to grading.

Several protocols of heparin-free dialysis are currently being used in routine practice, which may include saline flushes delivered at frequent intervals, requiring close monitoring by dialysis staff, or predilution, where a continuous infusion of saline is run to the dialyzer; the former procedure being the most commonly used. Taking into account this heterogeneity and in order to increase the external validity of the study results, it was decided to allow both types of saline infusions, which reflect real-life situations; however, accompanied with guidelines relative to the volume and output of saline infusion, in order to minimize the heterogeneity within the control group. To the best of our knowledge, the HepZero study is the first international, multicenter, randomized, controlled study aimed at comparing two techniques of heparin-free dialysis, including its gold standard, in two parallel groups, in an open-label design. It is sufficiently powered to first evaluate the non-inferiority of Evodial (which may allow a decrease in dialysis staff workload during heparin-free dialysis sessions) then its potential superiority versus the standard care of saline infusions, and may therefore have major clinical implications for patient care.

## Trial status

Trial status at the time of submission the 15^th^ January 2013: 245 patients recruited.

## Abbreviations

DSMB: Data and Safety Monitoring Board; PEI: Polyethyleneimine; RCA: Regional citrate anticoagulation; UF: Ultrafiltration.

## Competing interests

The authors declare that they have no competing interests relevant to the present manuscript, besides the disclosures presented below.

## Authors’ contributions

The authors are HepZero steering committee members as well as the study statistician (RF). They conceived of the study, participated in its design and coordination, and helped to draft the manuscript. All authors read and approved the final manuscript.

## Supplementary Material

Additional file 1 Appendix (supplementary material). Click here for file
