# Supplementary material for: Rationale and design of the HepZero study: a prospective, multicenter, international, open, randomized, controlled clinical study with parallel groups comparing heparin-free dialysis with heparin-coated dialysis membrane (Evodial) versus standard care: study protocol for a randomized controlled trial
Source: Trials. 2013 Jun 1;14:163. doi: 10.1186/1745-6215-14-163 (PMC3681640; doi:10.1186/1745-6215-14-163)
Supplement: Additional file 1 — Appendix (supplementary material). [file 1745-6215-14-163-S1.doc]

***Appendix (supplementary material)***

The following are the respective committee members in the HepZero trial:

Steering committee:

- Maurice Laville (Lyon, France)
- Patrick Rossignol (Nancy, France)
- Joan Fort Ros (Barcelona, Spain)
- Marc Dorval (Moncton, Canada)
- Frédérique Moreau and Nathalie Loughraieb (Gambro, Meyzieu, France)

Data Safety Monitoring Board:

- Dominique Joly (Paris, France)
- Claude Guérin (Lyon, France)
- Philippe Nguyen (Reims, France)

Centers, countries, investigators, institutional review boards:

| ***investigators:*** |  | **Institutional Review boards** |
| --- | --- | --- |
| *Study Site* | *Names* |  |
| **CHU Nancy Brabois**  Vandoeuvre  France | Pr Luc Frimat | **CPP Sud-Est III**  **69003 Lyon** |
| **CHU Lyon Edouard Herriot**  Lyon  France | Pr Laurent Juillard | **CPP Sud-Est III**  **69003 Lyon** |
| **CHU de Saint-Étienne - Hôpital Nord**  Saint-Priest en Jarez  France | Dr Damien Thibaudin | **CPP Sud-Est III**  **69003 Lyon** |
| **Vall d’Hebron Hospital**  Barcelona  Spain | Dr Juan Fort Ros | **CEIC**  **Vall d’Hebron Hospital** |
| **Hospital Universitari Germans Trias i Pujol**  Badalona  Spain | Dra Loreto Fernandez Lorente | **CEIC**  **Germans Trias i Pujol Hospital** |
| **Royal Liverpool University Hospital**  Liverpool  UK | Dr Michael Schulz | **NHS**  **NRES Committee Yorkshire & the Humber-Sheffield** |
| **Erasme Hospital**  Brussels  Belgium | Pr Joëlle Nortier | **Comité d’éthique**  **Université Libre de Bruxelles**  **Hopital Erasme** |
| **George Dumont Hospital**  Moncton New Brunswick  Canada | Dr Marc Dorval | **Comité d’éthique de la recherche**  **Centre hospitalier universitaire Dr-Georges-L.-Dumont** |
| **UMCG**  Groningen  Netherlands | Dr Casper Franssen | **MET**  **Universitair Medisch Centrum Groningen** |
| **Gdanski Uniwersytet Medyczny**  Gdansk  Poland | Prof. Bolesław Rutkowski | **Independent Bioethical Commission for scientific Research**  **Medical University of Gdansk** |
